# Model training across multiple breeding cycles significantly improves genomic prediction accuracy in rye (*Secale cereale* L.)

**DOI:** 10.1007/s00122-016-2756-5

**Published:** 2016-08-01

**Authors:** Hans-Jürgen Auinger, Manfred Schönleben, Christina Lehermeier, Malthe Schmidt, Viktor Korzun, Hartwig H. Geiger, Hans-Peter Piepho, Andres Gordillo, Peer Wilde, Eva Bauer, Chris-Carolin Schön

**Affiliations:** 1 Plant Breeding, TUM School of Life Sciences Weihenstephan, Technical University of Munich, Liesel-Beckmann-Str. 2, 85354 Freising, Germany; 2 KWS LOCHOW GMBH, Ferdinand-von-Lochow-Straße 5, 29303 Bergen, Germany; 3 Institute of Plant Breeding, Seed Science and Population Genetics, University of Hohenheim, Fruwirthstr. 21, 70599 Stuttgart, Germany; 4 Biostatistics Unit, Institute of Crop Science, University of Hohenheim, Fruwirthstr. 23, 70599 Stuttgart, Germany

## Abstract

**Key message:**

**Genomic prediction accuracy can be significantly increased by model calibration across multiple breeding cycles as long as selection cycles are connected by common ancestors.**

**Abstract:**

In hybrid rye breeding, application of genome-based prediction is expected to increase selection gain because of long selection cycles in population improvement and development of hybrid components. Essentially two prediction scenarios arise: (1) prediction of the genetic value of lines from the same breeding cycle in which model training is performed and (2) prediction of lines from subsequent cycles. It is the latter from which a reduction in cycle length and consequently the strongest impact on selection gain is expected. We empirically investigated genome-based prediction of grain yield, plant height and thousand kernel weight within and across four selection cycles of a hybrid rye breeding program. Prediction performance was assessed using genomic and pedigree-based best linear unbiased prediction (GBLUP and PBLUP). A total of 1040 S_2_ lines were genotyped with 16 k SNPs and each year testcrosses of 260 S_2_ lines were phenotyped in seven or eight locations. The performance gap between GBLUP and PBLUP increased significantly for all traits when model calibration was performed on aggregated data from several cycles. Prediction accuracies obtained from cross-validation were in the order of 0.70 for all traits when data from all cycles (*N*_CS_ = 832) were used for model training and exceeded within-cycle accuracies in all cases. As long as selection cycles are connected by a sufficient number of common ancestors and prediction accuracy has not reached a plateau when increasing sample size, aggregating data from several preceding cycles is recommended for predicting genetic values in subsequent cycles despite decreasing relatedness over time.

**Electronic supplementary material:**

The online version of this article (doi:10.1007/s00122-016-2756-5) contains supplementary material, which is available to authorized users.

## Introduction

Rye (*Secale cereale* L.) is a small grain cereal used for food, feed and in growing demands also for ethanol and biomethane production (Geiger and Miedaner 2009). Due to its ability to tolerate adverse growing conditions such as severe cold, drought or hostile soils rye is highly valuable for expanding cereal production to a wide range of agro-climatic conditions (Schlegel 2014). In contrast to other small grain cereals such as wheat, barley and oats, genetic progress in the cross-pollinated species rye is generated in selection schemes combining development of elite lines as hybrid components and population improvement through recurrent selection (Geiger 2007; Tomerius and Geiger 2001; Wilde 1996). Within each of two populations, the seed and the pollen parent pool, inbred lines are developed from crosses of elite parents with subsequent selfing, and selection candidates are evaluated for their combining ability as testcrosses. In the seed parent pool, promising selection candidates need to be transferred to a reliable cytoplasmic male sterility system before they can be crossed to a tester from the opposite pool. In the pollen parent pool, inbred lines need to carry efficient fertility restoration genes. As a consequence of high inbreeding depression, rye inbred lines are only selfed for a limited number of generations and exhibit substantial residual heterozygosity, compared to crops with an established doubled-haploid system as maize or barley. Because rye is mainly cultivated as a winter cereal, generation intervals are long and the development of hybrid components takes many years. Therefore, one major focus of rye breeding research lies on utilizing genomic tools to accelerate breeding progress.

The molecular toolbox of rye has constantly grown and enabled genome enhanced breeding during the last years. High-density genotyping platforms such as the Rye5k array (Haseneyer et al. 2011) and a custom 16k Infinium iSelect HD BeadChip (Illumina^®^) are available. A comprehensive expressed sequence tag (EST) resource was generated (Haseneyer et al. 2011) and whole genome sequencing is currently in progress (Bauer et al. 2015). While marker-assisted backcrossing as well as selection for individual genes with diagnostic markers have become routine applications, the efficiency of whole-genome based prediction (GP) in rye breeding populations still needs to be evaluated.

A key objective of GP is the accurate prediction of the genetic value of yet unphenotyped lines based on their DNA profile. In population improvement, essentially two prediction scenarios arise: (1) within breeding cycles, i.e., prediction of genetic values of progeny derived from the same or related crosses within the breeding cycle in which model training is performed and (2) across breeding cycles, i.e., prediction of consecutive generations of progeny generated from crosses with variable levels of relatedness to current genetic material. Various studies have reported prediction performance that encourages the implementation of genome-based prediction in breeding programs. In a wide range of crops, prediction accuracies ranged from intermediate to high (Lin et al. 2014; Zhao et al. 2015). Many of these studies were conducted on large biparental populations (Krchov et al. 2015), highly unbalanced historical data sets (Sallam et al. 2015) or closed populations employed in recurrent selection (Li et al. 2015) and their results are not directly transferable to advanced-cycle breeding populations as these populations have very different family structures, effective population size, allele frequency spectra, linkage disequilibrium and quality of phenotypes.

First promising results for genome-based prediction have been attained for rye by Bernal-Vasquez et al. (2014). They reported prediction accuracies obtained from cross-validation within selection cycles and years. These estimates must be considered as upper bound because of close familial relatedness and shared environmental conditions between the calibration and the validation data sets. It is the prediction of the genotypic value of selection candidates of the next cycles, from which the strongest impact of genome-based prediction can be expected. A study performed on data from two consecutive breeding cycles in sugar beet (*Beta vulgaris* L.) showed that across-cycle prediction accuracy depended on the trait under study and the authors pointed out that within-cycle prediction accuracy was not suited as indicator for the performance of across-cycle prediction (Hofheinz et al. 2012). For maize (*Zea mays* L.), prediction accuracies across subsequent cycles of selection were only slightly reduced for grain yield and dry matter content, compared to accuracies obtained with cross-validation within the same cycle when effects arising from population structure and choice of tester were modeled appropriately (Albrecht et al. 2014). In a study on five breeding cycles of bread wheat, Michel et al. (2016) reported a substantial decrease of prediction accuracy for three traits when predicting across instead of within selection cycles.

To investigate the factors influencing across-cycle prediction accuracy, we built a unique data set comprising high-precision phenotypes and high-density genotypes representing multiple interconnected rye breeding populations. We focused on three main objectives, (1) to comparatively assess the prediction performance of pedigree-based and genomic best linear unbiased prediction within and across breeding cycles, (2) to gain insight into the main components driving prediction performance across subsequent breeding cycles, and (3) to develop recommendations for model training to obtain maximum across-cycle prediction accuracies.

## Methods

### Genetic material

The genetic material used in this study consists of four data sets of advanced-cycle inbred lines (S_2_) from subsequent cycles (Cycle 1 to Cycle 4) of a commercial hybrid rye breeding program. The four data sets comprised a total of 1416 S_2_ lines for which up to ten generations of pedigree information was available. To represent each selection cycle by the same number of progenies, 260 S_2_ lines were randomly chosen from each of the four data sets, resulting in 1040 S_2_ inbred lines and all subsequent representations of results are based on these 1040 S_2_ lines representing progenies from 430 crosses of 203 parental lines. Genetic relatedness between the four data sets is given through a minimum of eight and a maximum of 21 common parental lines (Figure S1). On average, two inbred lines (min = 1, max = 24) were derived per cross, with 400 crosses yielding five or fewer inbred lines. To obtain testcross seed, each S_2_ line was crossed to two out of eight F_1_ pollen-sterile testers (T1–T8, see Table S1) showing different levels of relatedness. Testers represent a gametic sample of the complementary heterotic seed parent pool. Plant materials described in this study are proprietary to KWS LOCHOW GMBH.

### Phenotypic data analysis

Testcrosses of S_2_ lines were evaluated in seven or eight locations in the years 2009–2012, with several trial locations in Germany and one location in Poland. Within locations and separately for each tester, testcrosses were allocated to a series of trials laid out as α-lattice designs with two replicates on 5.5 m^2^ plots, connected by four elite hybrid checks. A general representation of the allocation of testers and locations within each of the four breeding cycles is given in Table S1. Locations and testers were confounded in Cycle 3, whereas the two testers shared one to two common locations in the other three cycles. In the following, the combination of location and tester is denoted location.tester. Testcross performance was evaluated for the traits grain dry matter yield (GDY, dt ha^−1^), plant height (PHT, cm), and thousand kernel weight (TKW, g), with TKW measured in all trials with one replication only. Phenotypic data were analyzed following a two-stage approach. In the first stage, adjusted entry means for genotypes (testcrosses of S_2_ lines) were calculated separately for each location and for each of the two testers by standard lattice analysis (Utz 2004). In the second stage, best linear unbiased estimates (BLUEs) of genotypes were calculated across testers and locations based on adjusted entry means obtained from the first stage using a mixed model including genotype as fixed effect and location.tester and genotype × location.tester interaction as random effects. Adjusted means from the first stage were weighted as described in method 1 of Möhring and Piepho (2009). Outlier detection was performed by consecutively detecting and removing outliers on the basis of maximum deviate residuals according to Grubbs (1950). For estimation of variance components the same models were used as for the calculation of adjusted means, except that genotypes were treated as random effects. Broad sense heritabilities (*h* ^2^) were calculated on a progeny-mean basis as described in method 1 of Estaghvirou et al. (2013). Calculations were performed using R (R Core Team 2015) or ASReml R (Butler et al. 2009).

### Genotyping

S_2_ lines were genotyped using a custom Rye 16 k Infinium iSelect HD BeadChip (Illumina, San Diego, CA, USA). Only high-quality SNPs with a GenTrain score ≥0.7 and a call rate ≥0.9 were used. SNPs with a minor allele frequency (MAF) < 0.01 or >10 % missing values were discarded, resulting in 10,416 useful SNPs. For 5607 SNPs the genetic map position was available (Figure S2A). Missing values of mapped SNPs were imputed based on flanking markers using Beagle (Browning and Browning 2009) and missing values of unmapped SNPs by sampling from marginal allele distributions using the synbreed R package (Wimmer et al. 2012). Linkage disequilibrium (LD) between marker pairs was calculated for genetically mapped markers as *r* ^*2*^ (Hill and Robertson 1968).

### Prediction methods

To predict the testcross performance of S_2_ lines we applied pedigree (PBLUP) and genomic (GBLUP) best linear unbiased prediction which differ in the variance–covariance structure used to model random testcross effects. The two models can be written as

$$ {\text{PBLUP}}:{\mathbf{y}} = {\mathbf{X}{\varvec{\beta }}} + {\mathbf{Zt}} + {\mathbf{e}} $$

$$ {\text{GBLUP}}:{\mathbf{y}} = {\mathbf{X}{\varvec{\beta }}} + {\mathbf{Zu}} + {\mathbf{e}} $$

where ${\mathbf{y}}$ is the vector of adjusted means from the second stage of the phenotypic analysis, ${\varvec{\beta}}$ is a vector of fixed effects containing four factor levels for selection cycle, ${\mathbf{X}}$ and ${\mathbf{Z}}$ are incidence matrices, assigning the adjusted means to fixed and random effects, respectively. In the PBLUP model ${\mathbf{t}}$ is the vector of random testcross effects, assumed to be normally distributed with ${\mathbf{t}}\,\sim \,{\text{N}}({\mathbf{0}},{\mathbf{K}}\sigma_{t}^{2} )$. ${\mathbf{K}}$ denotes the matrix of expected kinship coefficients calculated on the basis of pedigree information, with $\sigma_{t}^{2}$ being the testcross variance pertaining to the PBLUP model. Residuals ${\mathbf{e}}$ are assumed to be independent and normally distributed with ${\mathbf{e}}\,\sim \,{\text{N}}({\mathbf{0}},{\mathbf{I}}\sigma_{p}^{2} )$, where ${\mathbf{I}}$ denotes an identity matrix and $\sigma_{p}^{2}$ the residual variance. The expected kinship matrix $({\mathbf{K}})$ was calculated as ${\mathbf{K}}\, = \,0.5{\mathbf{A}}$, where ${\mathbf{A}}$ denotes the additive genetic relationship matrix calculated according to standard procedures (Lynch and Walsh 1998) implemented in the synbreed R package (Wimmer et al. 2012). Assuming a single seed descent selfing scheme, the dimensionality of the respective ${\mathbf{A}}$ matrix can be reduced by omitting the selfing steps when building the ${\mathbf{A}}$ matrix and modeling the diagonal element of individual *i* using $A_{ii} = \sum\nolimits_{S = 0}^{x} {\left( {\frac{1}{2}} \right)^{S} + A_{gh} } \left( {\frac{1}{2}} \right)^{x + 1}$, with $A_{ii}$ being the diagonal element of ${\mathbf{A}}$ for individual $i$, $x$ the number of selfing generations, and $A_{gh}$ being twice the kinship coefficient between the parents (*g* and *h*) of individual $i$ in generation S_0_, i.e., before selfing. For S_2_ lines derived from S_1_ plants we set $x = 1$. In the GBLUP model, random testcross effects ${\mathbf{u}}$ are assumed to be normally distributed with ${\mathbf{u}}\sim {\text{N}}({\mathbf{0}},{\mathbf{U}}\sigma_{u}^{2} )$. ${\mathbf{U}}$ denotes the realized kinship matrix calculated on the basis of the marker data (Habier et al. 2007), with $\sigma_{u}^{2}$ being the testcross variance pertaining to the GBLUP model. Residuals ${\mathbf{e}}$ are assumed to be independent and normally distributed with ${\mathbf{e}}\sim {\text{N}}({\mathbf{0}},{\mathbf{I}}\sigma_{m}^{2} )$, where $\sigma_{m}^{2}$ is the residual variance.

### Cross-validation schemes and prediction accuracies

**Fig. 1:**
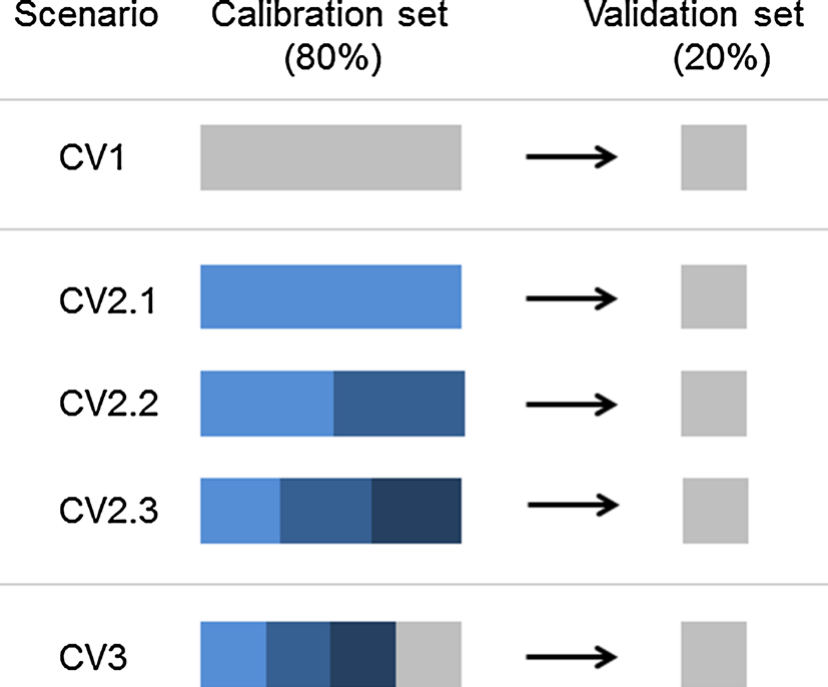
Cross-validation (CV) scenarios. *CV1* within-cycle CV with lines in calibration and validation from the same breeding cycle (*grey boxes*). Eighty percent of the lines from one cycle were used for calibration and twenty percent for validation. *CV2* across-cycle CV, where the calibration set comprised lines from other cycles than the validation set. CV2 calibration sets consisted of lines from one (CV2.1), two (CV2.2) or three (CV2.3) cycles (different shades of *blue*) with equal numbers of S_2_ lines from each cycle. *CV3* joint across- and within-cycle CV, where lines from all four cycles constituted the calibration set (*blue* and *grey boxes*), and lines from one of the cycles (*grey*) constituted the validation set. Lines from the validation set were not represented in the calibration set (color figure online)

Prediction accuracies were estimated applying different cross-validation scenarios (CV1-3, Fig. 1). Within-cycle (CV1) prediction accuracies were calculated by applying ten times replicated fivefold CV with random sampling using 80 % of the lines of a given cycle as calibration set (CS) and 20 % as validation set (VS) (Albrecht et al. 2011; Wimmer et al. 2012). In the across-cycle scenario (CV2), prediction accuracies were estimated using lines from one or multiple cycles as calibration set and lines from a different cycle as validation set. Three different scenarios were possible for CV2: in CV2.1 the calibration set was sampled from one, in CV2.2 from two, and in CV2.3 from three cycles. The third scenario (CV3) included randomly sampled lines from all four cycles in the calibration set except those lines included in the corresponding validation set. To allow a direct comparison of prediction accuracies, the allocation of genotypes to the validation sets was the same for all CV scenarios. When lines from multiple cycles constituted the calibration set, the same number of lines was sampled from each cycle. To evaluate the effect of sample size on prediction accuracy when aggregating data from multiple cycles, calibration set size in CV2.3 and CV3 was varied with *N*_CS_ = 208, 416, 624, and 832 (the latter only in CV3). CV2 and CV3 scenarios include all possible forward, as well as backward predictions in time. Variance components of PBLUP and GBLUP models were estimated by REML for each calibration set. For each CV scenario, prediction accuracy in validation set *v* was obtained by $r_{{\hat{Q}G_{v} }} = \frac{{\rho_{{\hat{Q}P_{v} }} }}{{\sqrt {h_{v}^{2} } }}$, where $\rho_{{\hat{Q}P_{v} }}$ denotes the predictive ability calculated as Pearson correlation coefficient between predicted ($\hat{Q}$) and observed ($P$) testcross values and $h_{v}^{2}$ the broad sense heritability for the respective trait and selection cycle from which validation set *v* was sampled (Dekkers 2007). To assess pairwise differences in accuracies between prediction models, a paired *t*-test was applied after Fisher's Z transformation.

### Analysis of germplasm

The relatedness of S_2_ lines in the calibration and validation set was analyzed for CV1 and for each of the 12 possible CV2.1 scenarios based on the average maximum realized kinship coefficient $(U_{\hbox{max} } )$ (Saatchi et al. 2011) derived from marker information. We calculated $U_{\hbox{max} ,i} = \hbox{max} (U_{ij} )$ with $U_{ij}$ being the realized kinship coefficient between line $i$ and line $j$ for $i\, \in \,VS$ and $j\, \in \,CS$. Averaging over S_2_ lines in the validation set resulted in a mean $\bar{U}_{\hbox{max} }$ value for the respective combinations of calibration and validation set. To detect hidden population substructure within breeding cycles, we performed a principal coordinate analysis (Gower 1966) based on Rogers’ distance (Rogers 1972) using the marker genotypes of the S_2_ lines.

## Results

### Germplasm structure

The 5607 mapped SNP markers were equally distributed across the genome with SNP numbers varying between 457 on chromosome 7R to 1091 on chromosome 5R (Figure S2A). LD decayed rapidly with 68 % of the marker pairs showing *r* ^*2*^ ≤ 0.2 within 1 cM (Figure S2B). As rye is an outcrossing species with low ancestral LD, a rapid decline of LD was expected in this data set of S_2_ lines derived from many crosses of at least partially unrelated parents. Heatmaps of the expected (**K**) and realized (**U**) kinship coefficients of the 1040 S_2_ lines are given in Figure S3. Within each cycle, family substructures are visible, but the principal coordinate analysis based on marker data indicated no major population substructure except for one large family in Cycle 1 (Figure S2C and D). $\bar{U}_{\hbox{max} }$ coefficients within cycles ranged from 0.27 to 0.29 and were substantially larger than $\bar{U}_{\hbox{max} }$ coefficients of the across-cycle scenarios ranging from 0.13 to 0.17.

### Phenotypic analyses

Testcross means for all traits differed significantly (*p* < 0.01) between breeding cycles (Table 1). For all traits and cycles, genotypic and genotype × location.tester (σ_g×l_^2^) variance components were highly significant (*p* < 0.01) and estimates of σ_g×l_^2^ were always smaller than the genotypic variance component. Trait heritabilities (*h* ^*2*^) on a progeny-mean basis were intermediate to high (Table 1). In Cycle 3, trait heritabilities were consistently lower compared to the other cycles.

**Table 1:** Testcross means with standard errors (S.E.), broad sense heritabilities (*h* ^*2*^) and variance components for grain dry matter yield (GDY), plant height (PHT) and thousand kernel weight (TKW) for four breeding cycles and *N* = 260 entries per cycle, respectively

Cycle^a^	GDY				PHT				TKW			
	Mean ± S.E.	*h* ^*2*^	σ_g_^2^ ^b^	σ_g×l_^2^ ^c^	Mean ± S.E.	*h* ^*2*^	σ_g_^2^	σ_g×l_^2^	Mean ± S.E.	*h* ^*2*^	σ_g_^2^	σ_g×l_^2^
1	90.8 ± 0.19	0.86	14.59	7.59	130.4 ± 0.26	0.91	35.73	11.75	36.4 ± 0.11	0.90	4.76	0.89
2	78.3 ± 0.23	0.86	15.77	3.77	126.7 ± 0.29	0.94	46.61	7.71	33.1 ± 0.11	0.80	6.71	4.31
3	81.6 ± 0.20	0.77	18.47	16.14	109.5 ± 0.28	0.89	38.09	23.10	37.0 ± 0.11	0.76	4.88	2.95
4	91.5 ± 0.23	0.83	22.96	16.51	124.3 ± 0.21	0.94	26.23	5.04	34.6 ± 0.10	0.87	4.85	2.09

^a^For number of locations, testers and year see Table S1

^b^Genotypic variance component

^c^Genotype × location.tester interaction variance component

### Within-cycle prediction accuracies

**Fig. 2:**
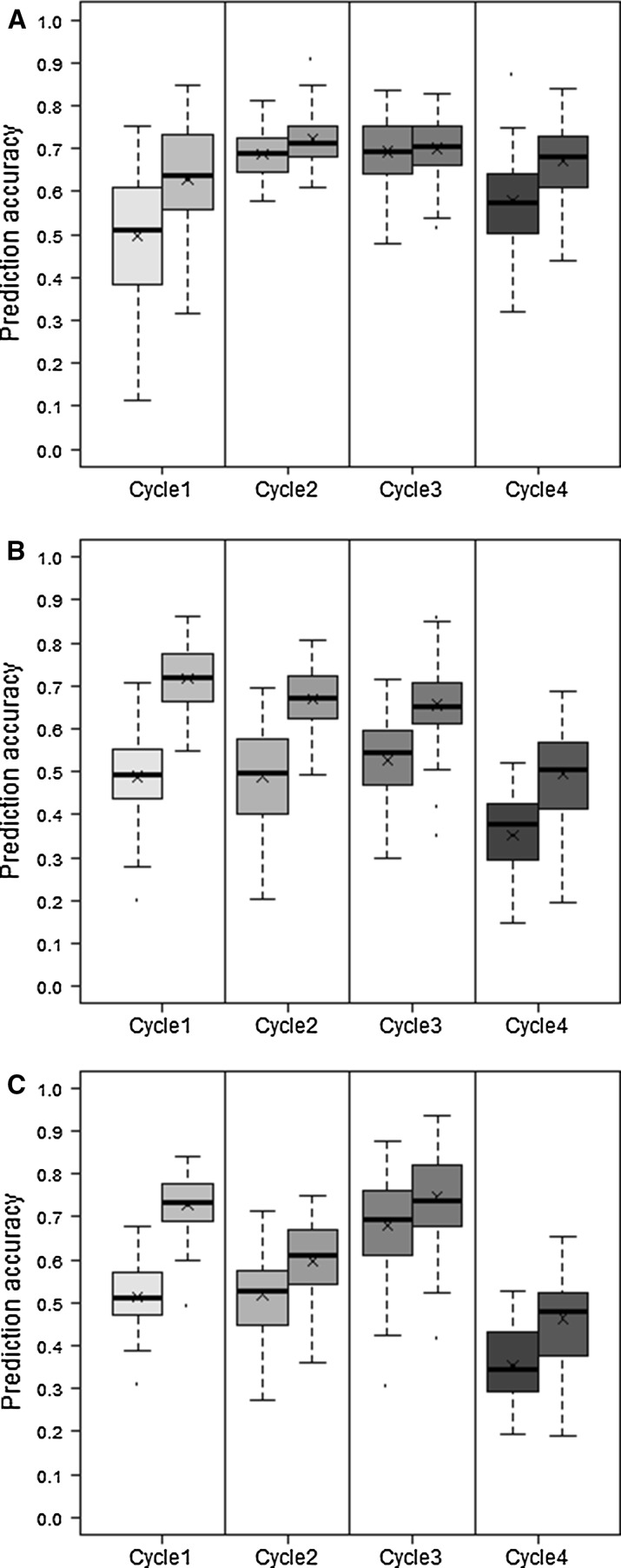
Within-cycle (CV1) prediction accuracies of four breeding cycles for **a** grain dry matter yield (GDY), **b** plant height (PHT) and **c** thousand kernel weight (TKW) obtained with PBLUP (*left*) and GBLUP (*right*). *Boxplots* show the median (*horizontal line*), mean (×), upper and lower quartile, and whiskers (*vertical bars*) of 10 × 5 fold cross-validation with random sampling and a constant calibration (*N* = 208) and validation set (*N* = 52) size. Points above and below the whiskers indicate values ±1.5 times the interquartile range

In CV1, calibration and validation sets originate from the same selection cycle, were crossed to the same two testers, and were evaluated in the same year. Within-cycle prediction accuracies for GDY obtained with PBLUP and GBLUP are shown in Fig. 2. GBLUP consistently outperformed PBLUP for all three traits. Averaged over the four cycles, mean prediction accuracies of GBLUP and PBLUP were highest for GDY (0.68 and 0.61), followed by TKW (0.63 and 0.52) and PHT (0.63 and 0.46). For GDY, the relative advantage of GBLUP over PBLUP was only marginal in Cycles 2 and 3. In contrast to Cycle 1 these two cycles comprised no large biparental family and had a higher average level of relatedness than Cycle 4.

### Across-cycle prediction accuracies

#### Single-cycle calibration sets

**Fig. 3:**
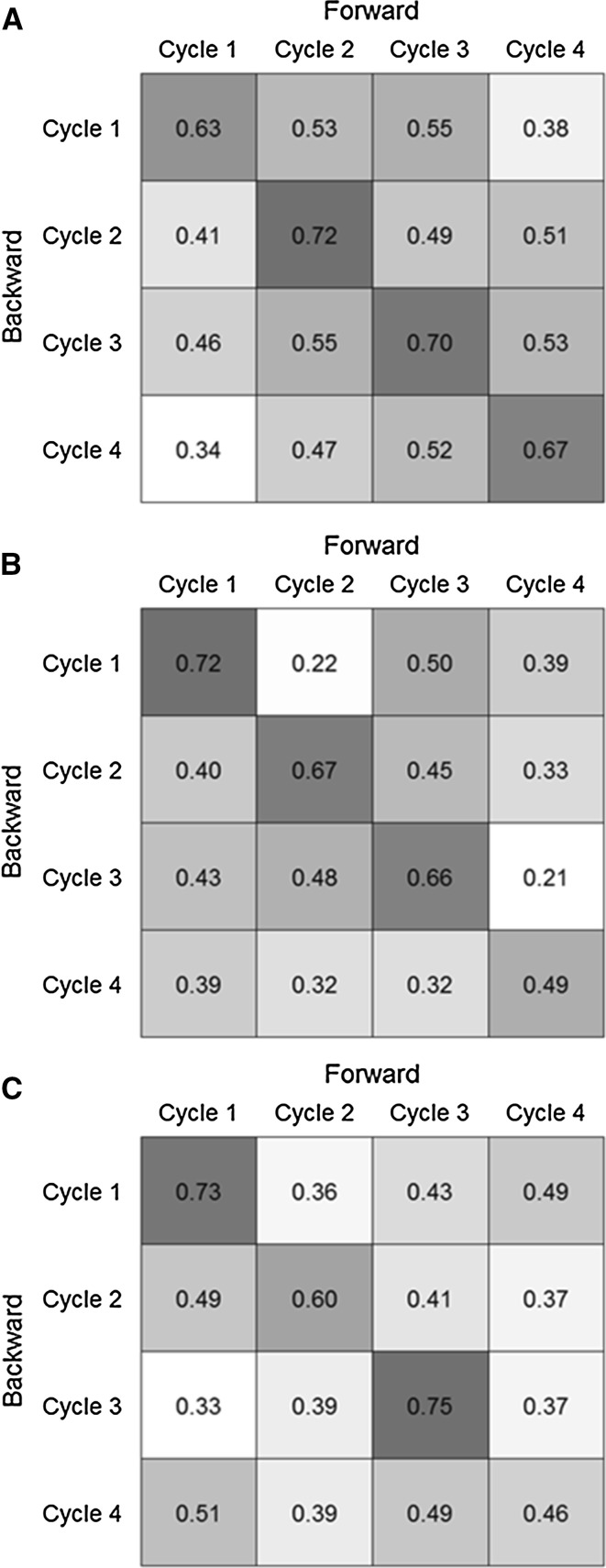
Within-(*CV1*, diagonal elements) and across-(*CV2.1* off-diagonal elements) cycle prediction accuracies for **a** grain dry matter yield (GDY), **b** plant height (PHT) and **c** thousand kernel weight (TKW) from GBLUP performing 10 × 5 fold cross-validation with constant calibration (*N* = 208) and validation set (*N* = 52) sizes. *Upper* (*lower*) triangular matrices constitute the forward (backward) across-cycle prediction direction

In CV2.1, the calibration and validation sets originate from different selection cycles, were crossed to different testers, and were evaluated in different years (Table S1). Averaged across the six possible single-cycle forward predictions in CV2.1 with sample size *N*_CS_ = 208, accuracies amounted to $\bar{r}_{{\hat{Q}G}} = \;0.50$ for GBLUP compared to $\bar{r}_{{\hat{Q}G}} = \;0.35$ for PBLUP. For PHT and TKW across-cycle prediction based on pedigree information was not possible with average forward prediction accuracies of 0.06 and 0.13, respectively. Genome-based prediction, on the other hand, yielded intermediate prediction accuracies of 0.35 for PHT and 0.40 for TKW. In all cases, genome-based prediction accuracies across cycles were smaller than within cycles except for TKW where some predictions involving Cycle 4 as calibration or validation set yielded slightly higher accuracies across than within cycles (Fig. 3).

**Fig. 4:**
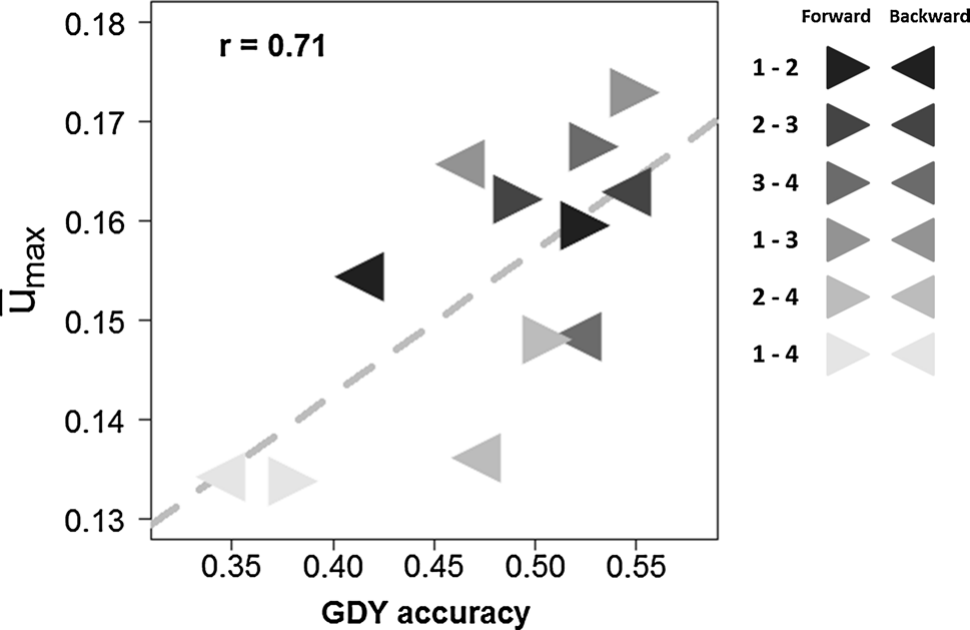
Across-cycle (CV2.1) prediction accuracies for grain dry matter yield (GDY) from GBLUP plotted against the average maximum kinship $\bar{U}_{\hbox{max} }$ (*r*, *p* < 0.01). Shaded triangles indicate cycles in calibration/validation set and forward/backward () prediction direction. Results are shown for all possible pairwise cycle combinations, with one cycle forming the calibration (*N* = 208) and one cycle the validation set (*N* = 52), respectively

The effect of relatedness of the calibration and validation set in the 12 possible single-cycle CV2.1 scenarios was assessed by calculating the Pearson correlation between $\bar{U}_{max}$ coefficients and the corresponding across-cycle prediction accuracies (Fig. 4). For GDY, a significant positive correlation (*p* < 0.01) was observed but it was mainly driven by the low relatedness and prediction accuracies of Cycle 1 and Cycle 4. For traits PHT and TKW correlations were not significant.

#### Multiple-cycle calibration sets

**Fig. 5:**
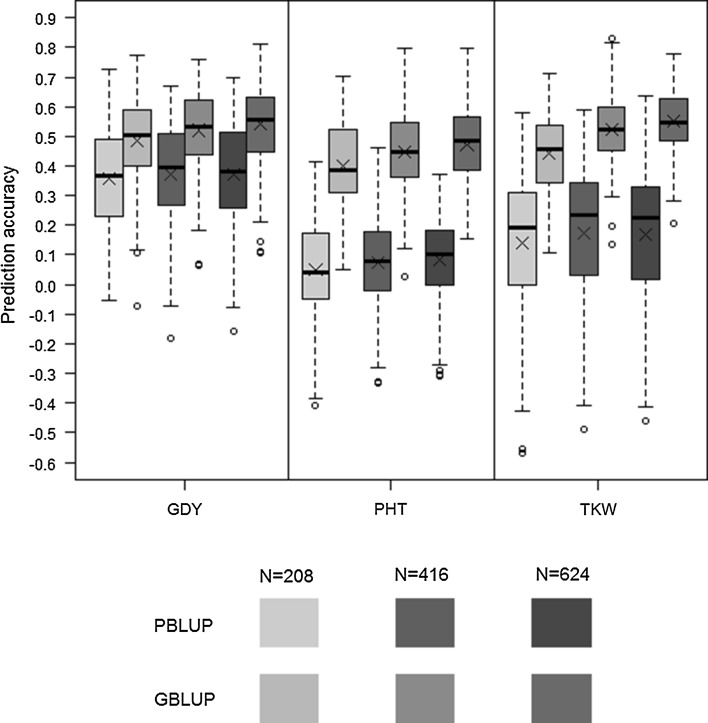
Across-cycle (CV2.3) prediction accuracies for grain dry matter yield (GDY), plant height (PHT), and thousand kernel weight (TKW) obtained with PBLUP and GBLUP with lines from three cycles forming the calibration set. *Boxplots* show the median (*horizontal line*), mean (×), upper and lower quartile, and whiskers (*vertical bars*) from 10 × 5 fold cross-validation with random sampling and increasing calibration set sizes of *N* = 208, 416 and 624 lines at constant validation set sizes of *N* = 52. For each pair of *boxplots* the *left* shows PBLUP and the *right* GBLUP. Points above and below the whiskers indicate values ± 1.5 times the interquartile range

To investigate the effect of combining lines from multiple cycles in the calibration set on prediction accuracies, we compared GBLUP model training based on calibration sets sampled from one, two or three cycles with a constant calibration set size of *N*_CS_ = 208. Mean prediction accuracies increased slightly for all three traits when sampling was performed from multiple cycles compared to sampling from one cycle only (Figure S5). PBLUP accuracies were substantially lower (values for CV2.1 see Figure S4 and for CV2.3 see Fig. 5) and showed a similar trend as GBLUP accuracies when predicting with multiple-cycle calibration sets.

A major advantage of combining data from multiple cycles for training the prediction model lies in the increased sample size of the aggregated calibration set as compared to data from the most recent preceding cycle only. By increasing the calibration set size from *N*_CS_ = 208 to 416 and 624 in CV2.3, we observed a clear positive trend of mean GBLUP prediction accuracies for all three traits (Fig. 5). At maximum sample size, average prediction accuracies increased to 0.54 for GDY, 0.55 for TKW and 0.47 for PHT. These accuracies were significantly higher (*p* < 0.01) compared to average accuracies obtained with single-cycle CV2.1 scenarios and similar as (GDY and PHT) or higher than (TKW) the best of the 12 single-cycle CV2.1 predictions given in Fig. 3. PBLUP predictions benefitted only slightly (TKW) or not at all (GDY, PHT) from the increase in sample size of the calibration set.

The effect of combining within- and across-cycle data for model calibration is shown in Table 2. While CV2.3 scenarios with *N*_CS_ = 624 could not reach average within-cycle accuracies for any of the three traits, prediction accuracies in CV3 outperformed those of CV1 with *N*_CS_ = 624 and even more so using the maximum possible population size *N*_CS_ = 832.

**Table 2:** Effect of calibration set (CS) sample size on prediction accuracies of GBLUP in the joint across- and within-cycle (CV3) scenario with lines from four cycles in the calibration set

CS sample size	GDY	PHT	TKW
208	0.60	0.52	0.56
416	0.64	0.60	0.64
624	0.68	0.65	0.68
832	0.70	0.69	0.70

Results for grain dry matter yield (GDY), plant height (PHT) and thousand kernel weight (TKW) were obtained by performing 10 × 5 fold cross-validation with constant validation set sizes (*N* = 52)

## Discussion

Evaluation of the potential of genome-wide prediction in plant breeding programs requires data sets that account for the specific properties of the employed selection schemes and populations. The data set employed here represents four advanced-cycle breeding populations of small effective population size with similar allele frequency spectra and extent of linkage disequilibrium. As the required time from recombination to the first performance test is five years, none of the four selection cycles comprised direct descendants of lines tested in earlier cycles. In contrast to recurrent selection on closed populations where pedigree relationships are reduced by half each generation, the relatedness of subsequent advanced-cycle plant populations depends on decisions made by the breeder with respect to the number of common parents and the influx of new genetic material. Thus, the relative advantage of genome- over pedigree-based prediction methods is difficult to assess theoretically and needs to be investigated with experimental data.

### Pedigree- and genome-based prediction across cycles

We showed that the performance gap between GBLUP and PBLUP increased significantly for all three traits when model training was performed on aggregated data from several selection cycles, indicating that accuracy of prediction will increase as information accumulates over time. For GDY, both prediction models (PBLUP and GBLUP) yielded intermediate prediction accuracies within and across cycles. As the average family size was rather small for many crosses, the moderate difference between PBLUP and GBLUP accuracies for GDY was not surprising. When decreasing the number of markers in the GBLUP model from 10,416 to 500, prediction accuracies were quite stable for GDY (data not shown) indicating that genome-wide relatedness and not so much marker-trait associations in specific genomic regions had a strong influence on prediction accuracy of this trait. This was supported by a significant correlation between the level of relatedness of the validation and calibration set with the corresponding across-cycle prediction accuracies for GDY in CV2.1 (Fig. 4).

For the two traits PHT and TKW, PBLUP and GBLUP prediction accuracies were intermediate to high within cycles but pedigree-based prediction averaged close to zero across cycles. We hypothesize that family-specific QTL with large or intermediate effects are segregating for the two traits. To support this hypothesis we compared marker effects estimated for the three traits in the full set of 1040 S_2_ lines using the Bayesian model BayesCπ (Habier et al. 2011) (Figure S6). For PHT and TKW more and larger marker-trait associations were detected than for GDY. We assume that pedigree-based prediction could model these effects within cycles based on close familial relationships, while this was not possible across cycles with more distantly related genetic material. On the other hand, the GBLUP model could capture some of these larger effects through LD that persisted in the across-cycle scenarios. Our hypothesis of different genetic architecture of GDY and PHT is supported by a study on genome-based prediction in a biparental rye population derived from two elite parents (Wang et al. 2014) where QTL based prediction of PHT performed quite similar to genome-wide prediction while for GDY there was a large difference in prediction accuracy between the two approaches. Findings from QTL analyses point in the same direction (Miedaner et al. 2012). As genomic data will accumulate over time it will be attractive to use these data not only for prediction of genetic values but also for inference on marker effects. The discussion on which statistical methods are appropriate for inference on marker effects has just started (Kemper et al. 2015; Kumar et al. 2016) and warrants further research.

When aggregating data across selection cycles, GBLUP prediction accuracy increased while PBLUP performance remained constant or increased only slightly (TKW). By increasing the sample size of the calibration set and modeling marker effects over several testers and years through data aggregation, not only an increase in mean prediction accuracy was achieved but also a slight reduction in prediction variance (Figure S5). Uncertainty of prediction is an important factor in optimization of breeding schemes but is often neglected in the discussion on the potential of genome-based selection. We conclude that the reduced variance of prediction is a further argument in favor of model training across several selection cycles.

### Factors influencing the accuracy of genome-based prediction across cycles

In across-cycle scenarios, the average maximum kinship of calibration and validation sets was about half that of within-cycle scenarios. As expected, a decrease in accuracy was found for CV2 compared to CV1 for both prediction models (PBLUP and GBLUP). When the breeding program advances, it can be assumed that selection cycles share fewer common ancestors. This was the case here with 8 (11) common parents of crosses for Cycle 4 and Cycle 1 (Cycle 4 and Cycle 2) compared to 18–21 common parents for the other pairwise combinations. This decrease in common ancestors over time was reflected in reduced kinship and significantly reduced accuracy when predicting lines from Cycle 4 with a model trained in Cycle 1. However, the relationship between average maximum kinship ($\bar{U}_{\hbox{max} }$) and prediction accuracy was intermediate for GDY (*p* < 0.01) and not significant for the other traits. This is in contrast to other studies where a strong linear relationship between average maximum kinship and prediction accuracy was found (Albrecht et al. 2014; Habier et al. 2010). In plant populations with influx of unrelated material, the average maximum kinship must be interpreted with caution as a predictor for accuracy even for complex traits like GDY. If the calibration set comprises a few entries that are highly related to many entries of the validation set, this will lead to high average maximum kinship but not necessarily to high prediction accuracy.

In several studies a decrease in prediction accuracy was reported when unrelated lines were added to the calibration set. These studies generally involved structured populations such as different animal breeds (e.g., Lund et al. 2014), different plant breeding programs (Lorenz and Smith 2015) or large biparental families (Riedelsheimer et al. 2013). In this study, we did not observe a decrease in prediction accuracy when aggregating data from several cycles which is expected from theory because unrelated or distantly related lines contribute almost nothing to prediction performance (de los Campos et al. 2013). Thus, we conclude that as long as selection cycles are connected by a sufficient number of common ancestors and prediction accuracy has not reached a plateau with respect to increases in sample size, aggregating data from several selection cycles is advisable for predicting the phenotypes of subsequent selection candidates despite decreasing relatedness over time. The set-up of optimum experimental designs to reach sufficient connectivity between breeding cycles for genome-based selection requires further research.

To separate the effect of increased precision of SNP effects due to i) larger sample size of the calibration set and ii) replication of alleles over years and testers, CV2 was performed with constant (*N*_CS_ = 208) and cumulated sample size of the calibration set (*N*_CS_ = 416, *N*_CS_ = 624). Mean prediction accuracies were very similar when sampling the same number of lines from one, two or three cycles, respectively. This indicates that the increase in prediction accuracy over cycles was mainly driven by an increase in sample size of the calibration set and that estimating marker effects based on testcrosses with more testers and evaluated in more years was of minor importance. With the given data it was not possible to separate the effects of across-cycle relatedness, genotype × year and genotype × tester interaction on prediction accuracy. We hypothesize that when averaging across two single-cross testers, specific combining ability effects can be assumed to be negligible. In addition, all S_2_ lines were evaluated in seven to eight locations in each year yielding very high progeny-mean heritabilities. Thus, we assume that genotype × location interactions within cycles could account to a large extent also for genotype × year interactions. The high precision of phenotypic data in our study might explain some of the discrepancies to studies on genome-based prediction in self-pollinating crops where merging data sets from subsequent progeny sets was rarely advantageous (e.g., Sallam et al. 2015). In self-pollinating crops, populations employed in model training frequently represent highly unbalanced historical data sets with many lines phenotypically evaluated at low intensity enhancing prediction accuracy only marginally.

The effect of sample size and replication on GBLUP prediction accuracy was investigated in a simulation study by Lorenz (2013) and a high degree of flexibility in the allocation of the two factors was observed. For experimental plant populations of small effective population size it was also shown that prediction accuracy could not be increased beyond a certain level despite increases in sample size (Albrecht et al. 2011; Jan et al. 2016). In this study, GBLUP predictive ability for GDY increased steadily till a sample size of about 800 S_2_ lines was reached (Figure S7). Thus, our data provide an excellent base for investigating the effect of allocation of resources for maximizing selection gain from genome-based selection per unit time and budget.

### Implementation of genome-based prediction in hybrid rye breeding

Mean prediction accuracies found in this study were greater 0.47 for all traits when aggregating data across three independent cycles (*N*_CS_ = 624, CV2.3) and could be increased to 0.69–0.70 in CV3 (*N*_CS_ = 832). Based on these results we conclude that genome-based prediction will be an important instrument in hybrid rye breeding to increase selection gain. How to implement genome-based prediction with maximum efficiency requires further research. The data employed here were taken from the first stage of a multi-stage selection scheme with strong priority on precision phenotypes and risk prevention in first selection steps (Wilde 1996). Such a selection scheme leads to slightly lower expected selection gains and to lower variance in gains in comparison to scenarios with higher selection but lower testing intensity. Implementation of genome-based prediction will require breeders to revisit their decisions on optimal allocation of resources. It can open new opportunities such as (1) to reduce cycle length from actual five years to four or even three years, (2) to change from phenotypic selection to more accurate indices combining both genomic and phenotypic information, and (3) to make full use of the genetic variance segregating in a selection scheme based on early testing of partially inbred families. On the other hand including genome-based prediction will require a more sophisticated management and design of crosses and familial structures than selection on phenotypes alone. A thorough investigation of resource allocation to phenotyping and genotyping is mandatory to maximize short- and long-term gain from selection. Insights derived from this study provide an excellent starting point for optimization of breeding schemes integrating genome-based prediction in hybrid rye breeding. The magnitude of prediction accuracies found is encouraging, suggesting that genomic prediction in rye is a worthwhile endeavor.

## Conclusion

We assessed the prediction performance of pedigree- and genome-based prediction within and across four breeding cycles of a hybrid rye program and found that the relative advantage of GBLUP over PBLUP increased significantly when model training was performed on aggregated data from several selection cycles. We conclude that as long as selection cycles are connected by a sufficient number of common ancestors and prediction accuracy has not reached a plateau with respect to increases in sample size, aggregating data from several preceding cycles is advisable for predicting phenotypes of selection candidates despite decreasing relatedness over time. Implementation of genome-based prediction will open new opportunities such as reducing selection cycle length and making full use of the genetic variance in each cycle. On the other hand, it will require a more sophisticated management and design of crosses and familial structures than selection on phenotypes alone. As genomic and phenotypic data will accumulate over time they will not only be useful for prediction of phenotypes but also for inferences on marker effects and genomic regions contributing to expression of quantitative traits.

### Author contribution statement

CCS, EB, PW, and AG designed the study. MSchm, AG and PW coordinated the field trials and phenotyping. VK, MSchm and EB coordinated marker development and genotyping. HJA, MSchö and CL analyzed the data. CCS, MSchö, and EB wrote the manuscript. All authors discussed and interpreted results and read and approved the final manuscript.

## Electronic supplementary material

Below is the link to the electronic supplementary material.

Supplementary material 1 (PDF 794 kb)
