# Supplementary material for: Model training across multiple breeding cycles significantly improves genomic prediction accuracy in rye (Secale cereale L.)
Source: Theor Appl Genet. 2016 Aug 1;129(11):2043–53. doi: 10.1007/s00122-016-2756-5 (PMC5069347; doi:10.1007/s00122-016-2756-5)
Supplement: Supplementary file 1 — Supplementary material 1 (PDF 794 kb) [file 122_2016_2756_MOESM1_ESM.pdf]

Supplementary material

Table S1 Allocation of testers (T) and locations (L) within the four cycles.

Location	Cycle 1 Tester		Cycle 2 Tester		Cycle 3 Tester		Cycle 4 Tester	
L-01		T2		T4		T6		T8
L-02	T1	T2	T3	T4	T5		T7	
L-03	T1		T3		T5			
L-04	T1		T3	T4				
L-05	T1							
L-06		T2		T4				T8
L-07		T2		T4		T6		T8
L-08			T3		T5		T7	
L-09					T5			
L-10						T6		
L-11						T6		
L-12							T7	
L-13							T7	T8
L-14							T7	

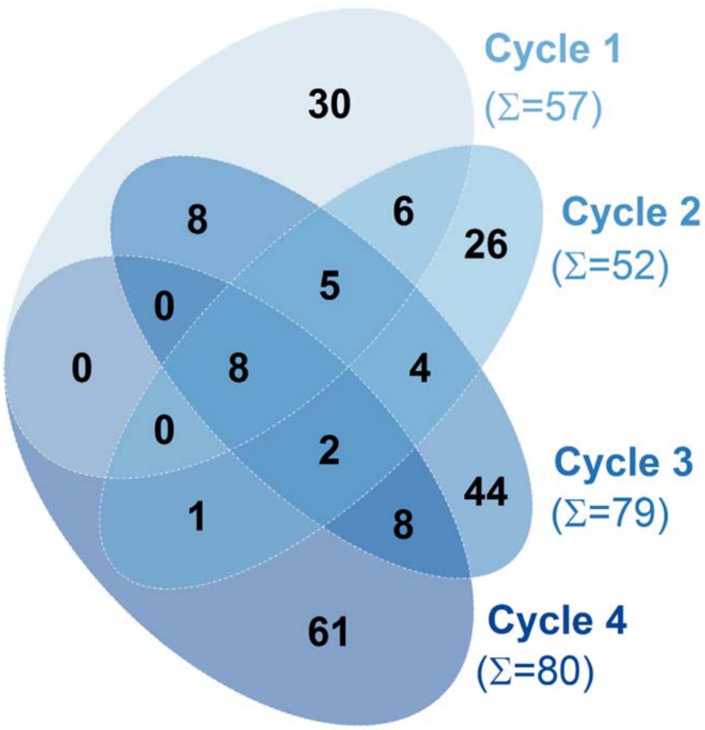

Figure S1 Venn diagram of 203 parental lines from which the 1,040 S_2 lines tested in four breeding cycles were derived. The numbers in brackets indicate the total number of ancestors per cycle including duplicated lines.

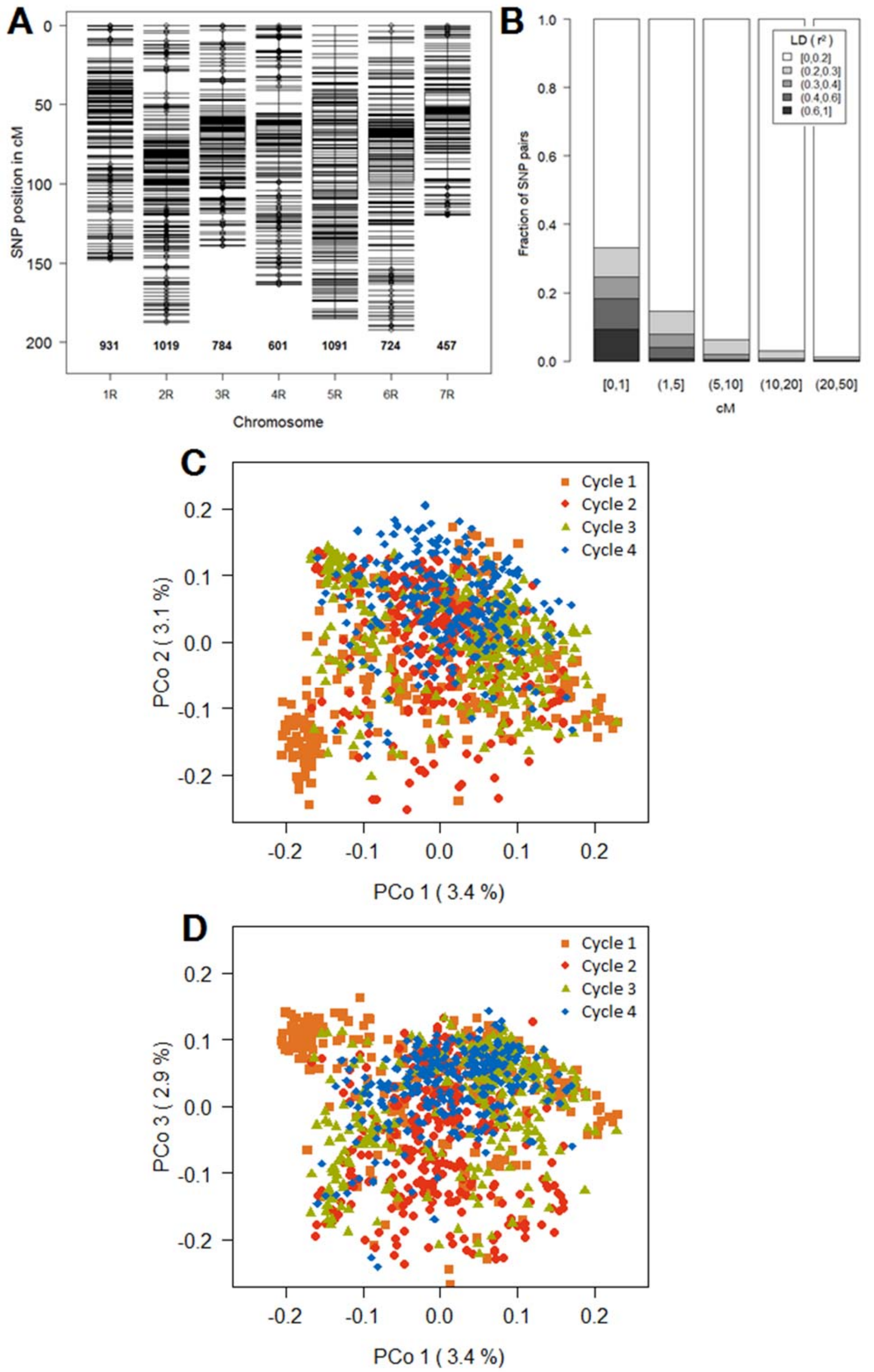

Figure S2 Linkage disequilibrium and population structure. **A**) Distribution and genetic position of 5,607 SNPs in the rye genome (Schmidt et al., unpublished). **B**) Linkage disequilibrium (LD) between pairs of markers for 5,607 SNPs exhibiting significant LD ($p < 0.05$) within chromosomes as a function of genetic distance in cM for 1,040 S_2 lines. **C**) Principal coordinates (PCo) 1 and 2 with S_2 lines colored according to their affiliation to one of the four breeding cycles. Numbers in brackets indicate the percentage of variance explained by the principal coordinates. **D**) Principal coordinates 1 and 3 with S_2 lines colored according to their affiliation to one of the four breeding cycles.

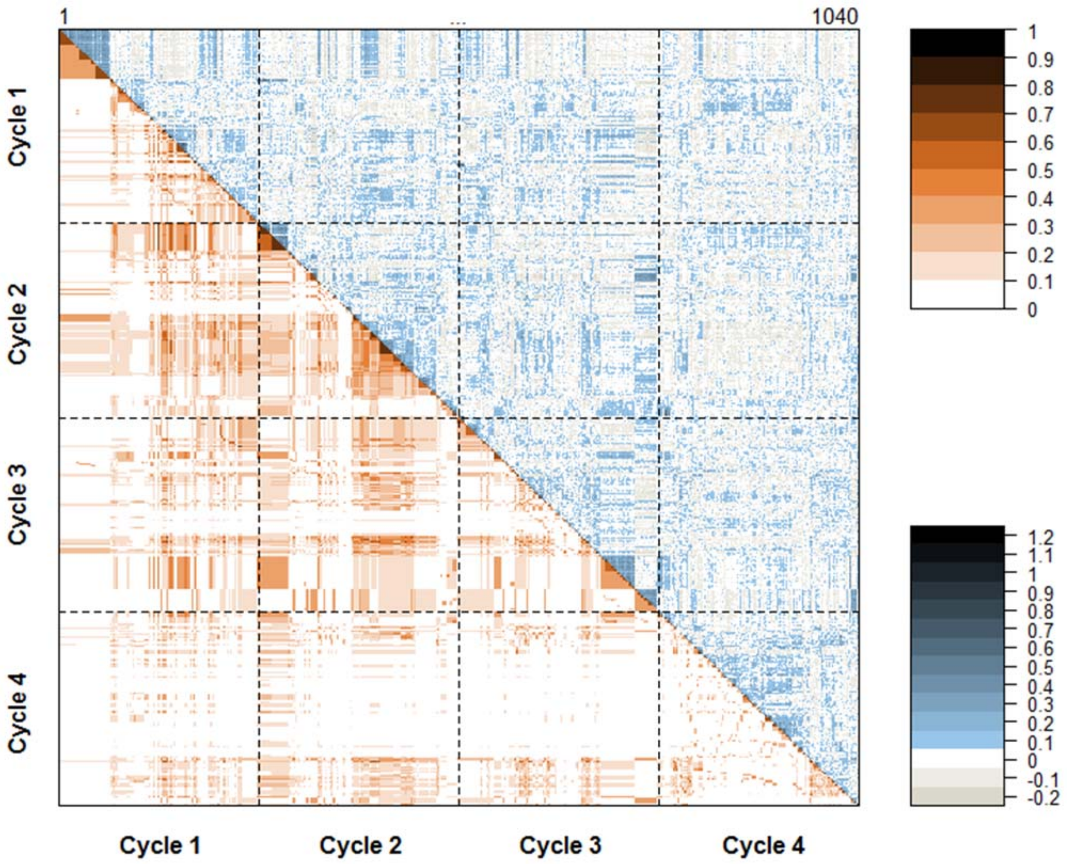

Figure S3 Expected (lower triangular matrix) and realized kinship (upper triangular matrix and diagonal elements) of 1,040 S_2 lines. Dashed lines separate the four breeding cycles. Heatmaps on the right indicate the respective kinship level.

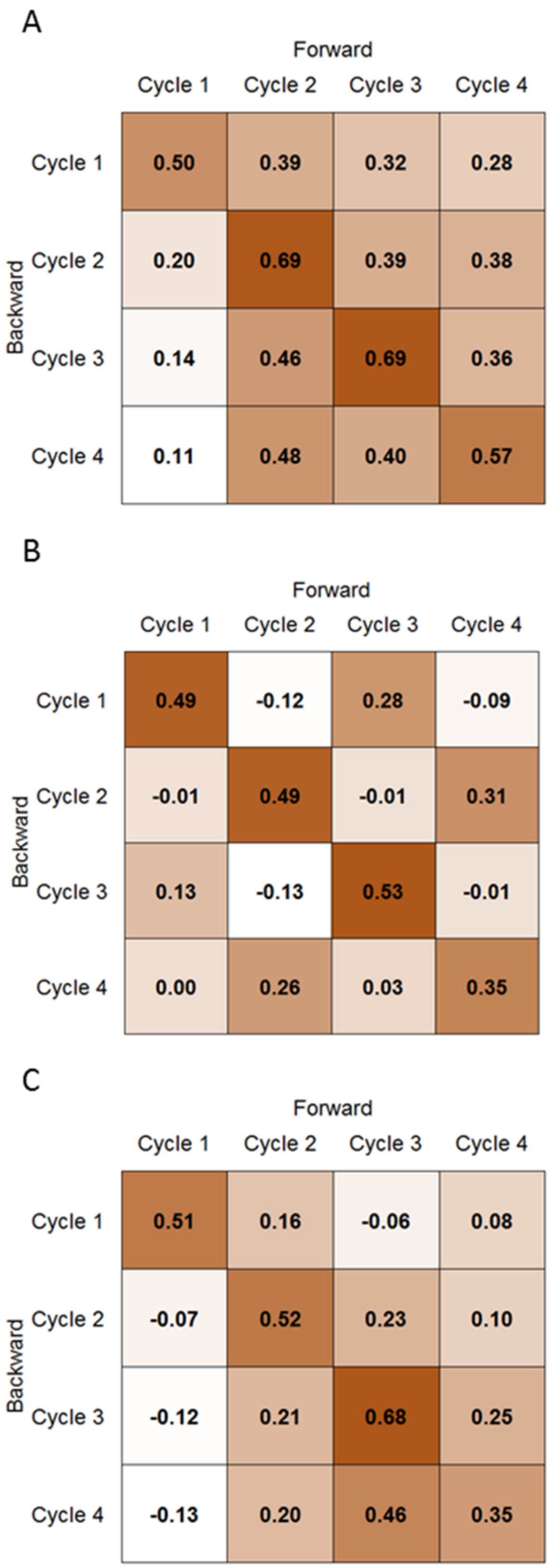

Figure S4 Within- (CV1, diagonal elements) and across- (CV2.1, off-diagonal elements) cycle prediction accuracies for **A**) grain dry matter yield (GDY), **B**) plant height (PHT) and **C**) thousand kernel weight (TKW) from PBLUP performing 10 × 5 fold cross-validation with constant calibration (N = 208) and validation set (N = 52) sizes. Upper (lower) triangular matrices constitute the forward (backward) across-cycle prediction direction.

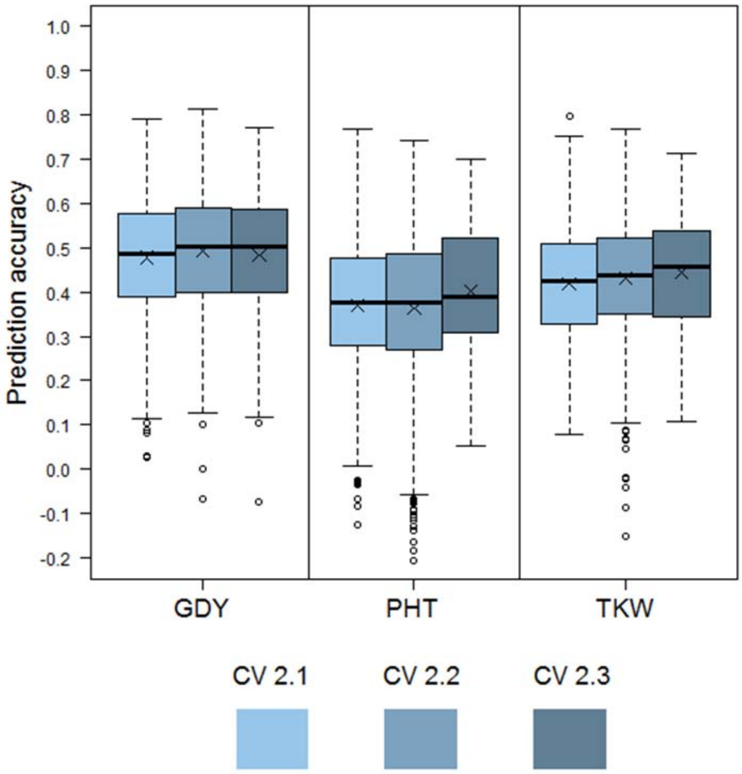

Figure S5 Across-cycle (CV2) prediction accuracies of GBLUP for grain dry matter yield (GDY), plant height (PHT) and thousand kernel weight (TKW) using all possible combinations of lines from one (CV2.1), two (CV2.2) or three (CV2.3) cycles forming the calibration sets and lines from a single-cycle the validation set. Boxplots show the median (horizontal bar), mean (×), upper and lower quartile, and whiskers (vertical bars) of 10 × 5 fold cross-validation with random sampling and a constant calibration (N = 208) and validation set (N = 52) size. Points above and below the whiskers indicate values ± 1.5 times the interquartile range.

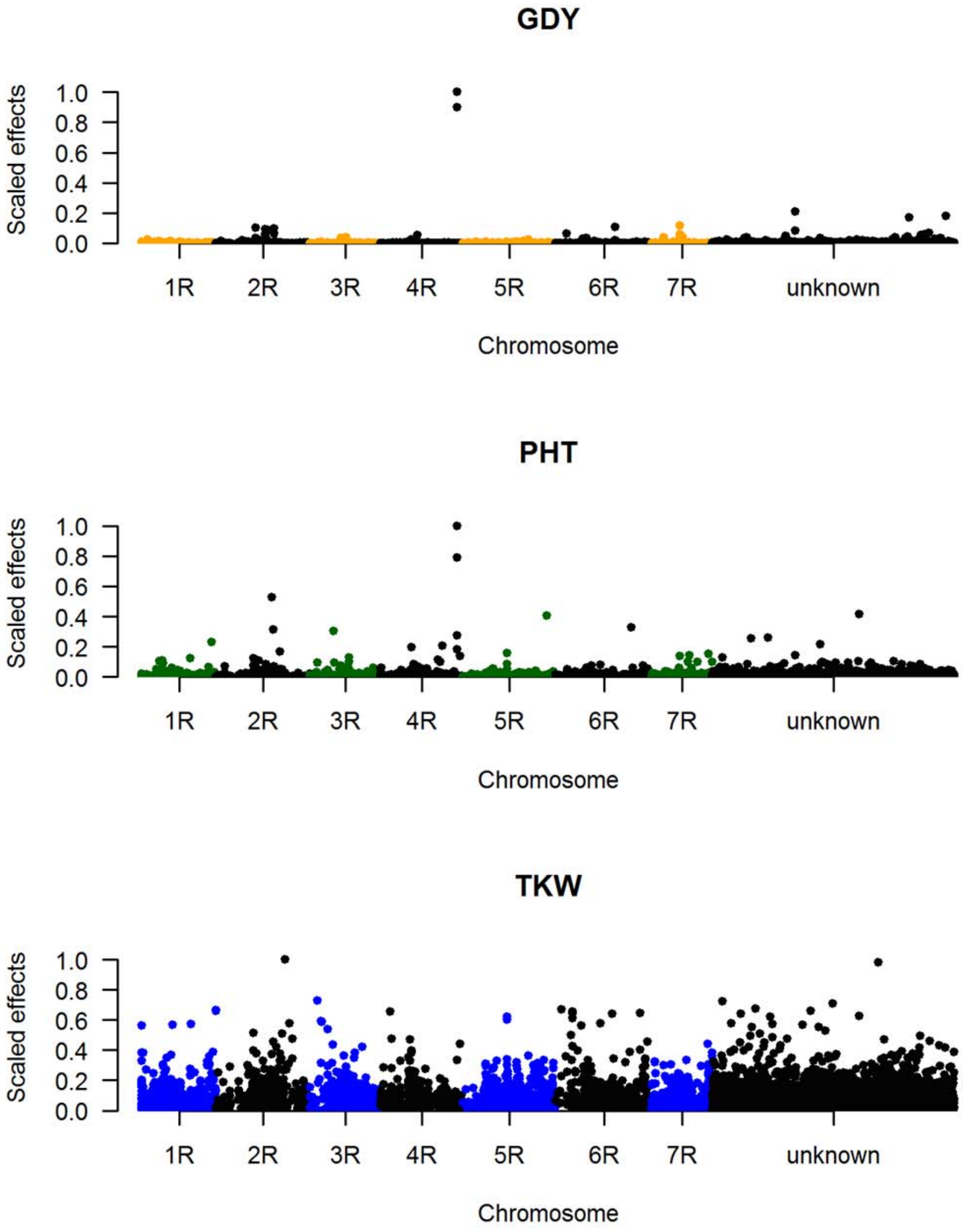

Figure S6 Genome-wide plot of SNP effects from BayesCπ. SNP effects were estimated with the variable selection method BayesCπ (Habier et al. 2011) as described by (Lehermeier et al. 2014) and plotted along the rye chromosomes 1R to 7R for the traits grain dry matter yield (GDY), plant height (PHT) and thousand kernel weight (TKW). For each trait the SNP effects were scaled from 0 to 1. SNPs with no genetic map position are labeled "unknown".

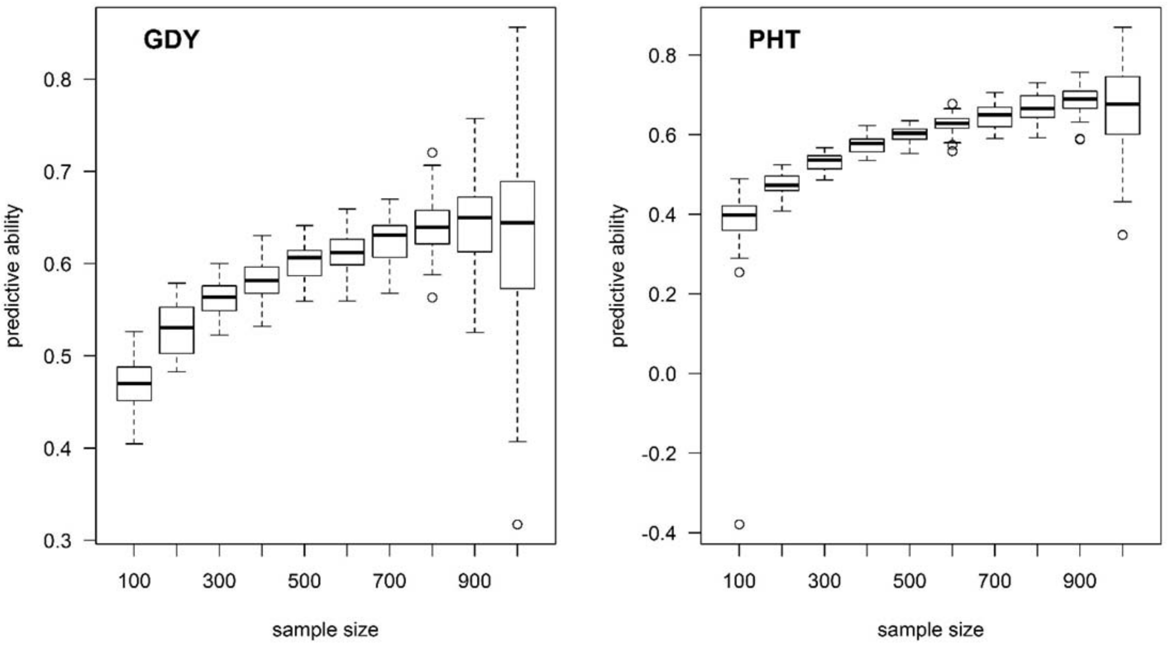

Figure S7 Predictive abilities for grain dry matter yield (GDY) and plant height (PHT) as a function of increasing sample size in the calibration set (N_{CS}) and decreasing sample size ($1,040 - N_{CS}$) in the validation set. Sampling of the calibration and validation sets from the 1,040 S_2 lines was performed in steps of 100 and repeated 50 times for a given N_{CS}. Boxplots show the mean (horizontal bar), upper and lower quartile, and whiskers (vertical bars) of the 50 replications. Points above and below the whiskers indicate values ± 1.5 times the interquartile range. Here, predictive abilities instead of accuracies are given, since accuracies could not be estimated properly as lines from several cycles with different heritabilities formed the validation set. The observed increase in variation of predictive ability with increasing sample size of the calibration set can be explained by an associated decrease of validation set size leading to larger variation as also observed for example by Erbe et al. (2010).

References:

Erbe M, Pimentel ECG, Sharifi AR, Simianer H (2010) Assessment of cross-validation strategies for genomic prediction in cattle. Proceedings of the 9th World Congress on Genetics Applied to Livestock Production (WCGALP), 1-6 August, Leipzig, Germany, p 129

Habier D, Fernando RL, Kizilkaya K, Garrick DJ (2011) Extension of the bayesian alphabet for genomic selection. BMC Bioinformatics 12:186

Lehermeier C, Krämer N, Bauer E, Bauland C, Camisan C, Campo L, Flament P, Melchinger AE, Menz M, Meyer N, Moreau L, Moreno-González J, Ouzunova M, Pausch H, Ranc N, Schipprack W, Schönleben M, Walter H, Charcosset A, Schön C-C (2014) Usefulness of multiparental populations of maize (*Zea mays* L.) for genome-based prediction. Genetics 198:3-16
